# Oral administration of tannins and flavonoids in children with acute diarrhea: a pilot, randomized, control-case study

**DOI:** 10.1186/s13052-018-0497-6

**Published:** 2018-06-04

**Authors:** Marina Russo, Vincenzo Coppola, Eleonora Giannetti, Roberta Buonavolontà, Antonio Piscitelli, Annamaria Staiano

**Affiliations:** Department of Translational Medical Science, Section of Pediatrics, “Federico II” University of Naples, Via S. Pansini, 5, 80131 Naples, Italy

**Keywords:** Acute gastroenteritis, Treatment, Tannins, Flavonoids

## Abstract

**Background:**

AG is the most common cause of pediatric consultations among children between 2 and 5 years of age and it still leads to high mortality and morbidity. Its management is based on rehydration therapy, but this treatment is not effective in reducing duration of diarrhea. For this reason, other safer and less expensive interventions, which could be added to oral rehydration therapy, are of great interest.

**Methods:**

A pilot, randomized, case-controlled trial was conducted in 60 children affected by AG (< 7 days) with mild-moderate dehydration, according to WHO recommendations, from1 year to 17 years old. Patients were divided into 2 Groups: Group 1 consisting of 30 children treated with Actitan F and standard oral rehydration (SOR); Group 2 consisting of 30 children who received only SOR. Both groups received treatment for seven days, respectively. Patients of Group 1 stopped for their own choice, SOR after the first 24 h and continued only with Actitan F.

**Results:**

After 24 h of treatment, the median number of stools was 3.5 for Group 1, and 4 for Group 2. In Group 1 the difference between the number of stools at baseline (*n* = 5) and after 24 h of treatment (*n* = 3.5) was significant (*p* < 0.0001). At the end of treatment, the median duration of diarrhea in Group 1 was 5 days, compared with 4 days in the Group 2, this difference was not statically significant (p 0.48).

**Conclusions:**

Oral administration of Actitan F associated with SOR seems safe and effective treatment in shortening the duration of AG in children. Further studies confirming these data are needed.

**Trial registration:**

NCT03356327 (retrospectively registered).

## Background

Acute gastroenteritis (AG) is the most common cause of pediatric consultations among children between 2 and 5 years of age [1]. AG in children still leads to high mortality and morbidity [2].According to current European guidelines, the main treatment for AG is the oral rehydration with anhypo-osmolar solution and regular feeding without dietary changes, including milk consumption. However, these treatment, does not reduce severity and duration of symptoms. Considering the burden of AG to children and the high cost for healthcare system, effective and less expensive interventions, that could be added to the effect of oral rehydration therapy, are of great interest [3].

According to the European Society of Gastroenterology and Hepatology (ESPGHAN) guidelines for diarrhoea [4], probiotics, with evidence of efficacy such as *S. boulardii* and *L. rhamnosus GG*, can be considered as a supplement to hypo-osmolar solution. It is known that probiotics reduce the mean duration of diarrhoea by about 24 h, but their efficacy on stool amount is not described [4]. Although the grade of recommendation is very strong, quality of evidences is still low [5]. Other preparations used for treatment of diarrhoea include oral administration of immunoglobulins, antiperistaltic and antisecretory agents, such as various preparations of bismuth subsalicylate, cholestyramine and loperamide [6–10]. Use of antiperistaltic drugs is not effective and can cause serious side effects in children, including lethargy, seizures, Reye’s syndrome, paralytic ileus and respiratory depression [11–13]. Use of loperamide, one of the most common antiperistaltic agents, is not recommended in young children and infants.

In conclusion, an ideal antidiarrheal agent should have a high index of safety even when used without close professional supervisions, and should be compatible with oral rehydration solutions, treating effectively diarrhea of any etiology with moderate cost. During last years, several randomized and controlled studies showed the efficacy of tannin-rich carob pod for the treatment of acute-onset diarrhea [10]. In 2003,Subbotina et al. demonstrated that tormentil root extract, which has high content of tannins, reduces the duration of rotavirus diarrhea compared with the control group in a pediatric population (*P* < 0.0001) [5].They also demonstrated that in the group treated with tormentil root extract, 8 of 20 children (40%) were diarrhea-free 48 h after admission to the hospital, compared with 1 of 20 in control group (5%, *P* < 0.0001) [5].

In this pilot study we aimed to evaluate the efficacy and the compliance of a treatment with Actitan-F, a natural molecular complex of tannins (from Agrimony and Tormentil) and flavonoids (Chamomile), associated with SOR, in a pediatric population of children affected by AG.

## Methods

We included 60 children (mean age: 3.1 yrs., range 0.3-12 years) with a diagnosis of AG, referred between April and July 2017 to the Department of Translational Medicine, section of Pediatric, University of Naples Federico II.

Patients enrolled were children from 3 months to 12 years old, with a diagnosis of acute diarrhoea appeared less than 7 days before the admission, capability to oral rehydration, mild to moderate dehydration. Patients with diarrhoea over 7 days, serious somatic pathology and severe dehydration were excluded.

The study was approved by the Institutional Review Board of the University of Naples “Federico II” with the protocol number 25/17. At admission, written informed consent was obtained from participants’ parents and from all patients older than 10 years. At first visit, a medical history was collected by one of the authors and all patients underwent clinical evaluation, including body weight and body temperature.

Frequency of bowel movements, stool consistency measured through the Bristol Stool Form Scale (BSFS) and other associated gastrointestinal symptoms, including nausea, vomiting, abdominal pain and rectal bleeding, were accurately recorded. The BSFS is the most commonly standardized instrument used to rate stool consistency in children [14]. On admission, the degree of dehydration was clinically determined for each patient, based on WHO recommendations and data were recorded on a scale from 1 to 3 (1 for mild or < 5%; 2 for moderate or 5 to 10%; 3 for severe or 10% and more) [15].

**Fig. 1:**
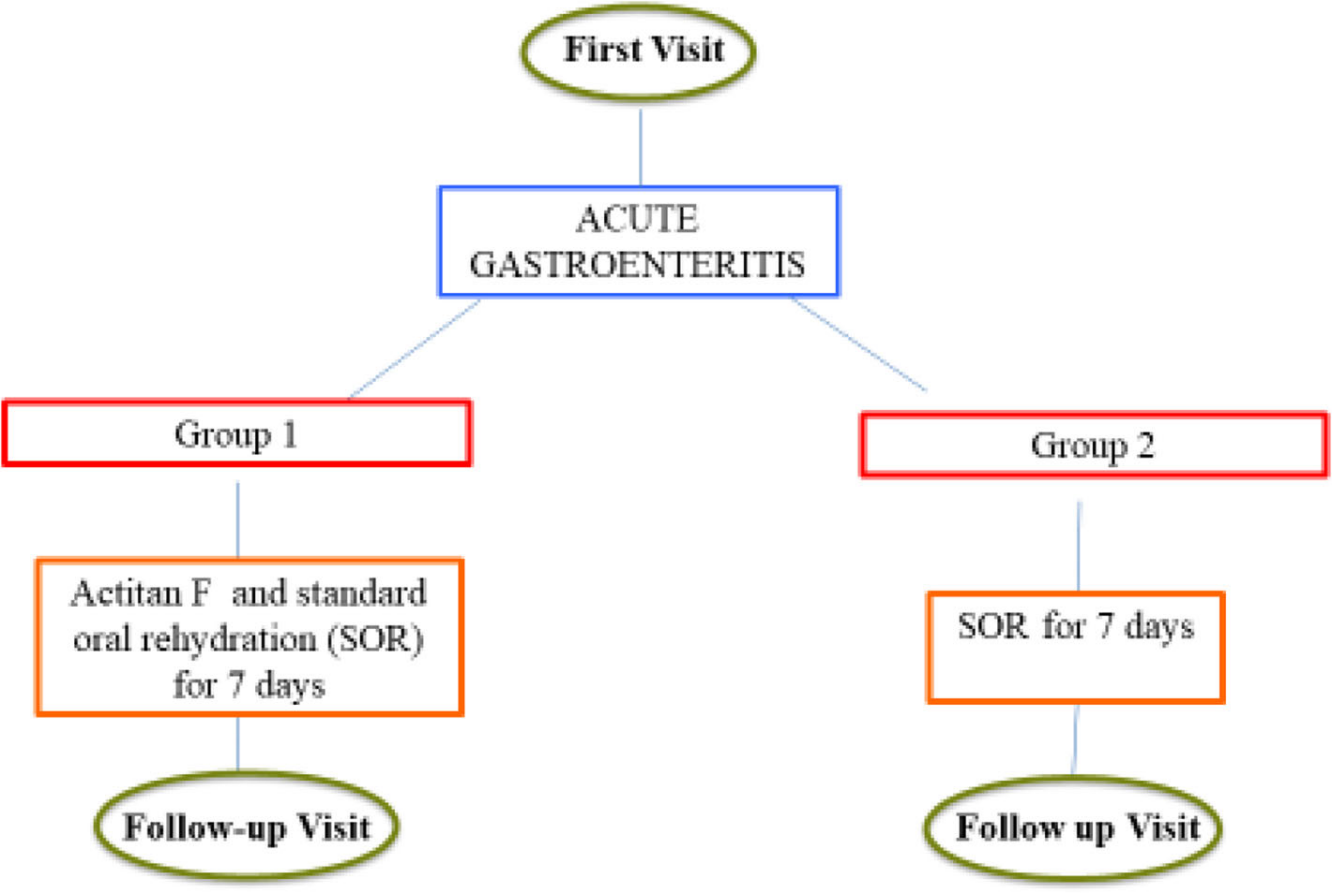
Flow-chart of the study

All enrolled children were randomly divided into two groups: Group 1 was treated with Actitan F and standard oral rehydration (SOR) and Group 2 was treated with SOR only ad libitum for 7 days (Fig. 1) SOR is a reduced osmolarity oral solution (50/60 mmol/L Na), which is the first line therapy recommended by ESPGHAN guideline for Acute Diarrhea [16]. Actitan F, instead, was orally administrated at dose of 1 sack every 4 h, maximum 4 sacks/day for 7 days. Caregivers were instructed to administer the daily dose after mixing the contents of the sachet with a small amount of water. The study products used in this trial were donated by Aboca® Società Agricola SpA., Località Aboca, 20, 52,037 Sansepolcro (AR) – Italy.

At home, all parents had to fulfil a daily diary to record number and consistency of stools, presence of fever, vomiting and children compliance with the therapy. During the final visit, scheduled after 7 days, the interim history was assessed, daily diaries were reviewed and discussed, and a physical evaluation was performed.

### Outcomes

The primary outcome was to evaluate the efficacy of a treatment with Actitan-F, a natural molecular complex of tannins (from Agrimony and Tormentil) and flavonoids (Chamomile) added to SOR compared to SOR in a paediatric population of children affected by AG.

The secondary outcome was to assess safety, tolerability and compliance of the study products for short-term treatment.

### Endpoints

The primary endpoint was the duration of diarrhoea, defined as the number of stools after 24 h of treatment or the time needed to normalize number and consistency of stools (compared with the period before the onset of diarrhoea). Secondary endpoint were the evaluation of vomiting, body weight, possible need of hospitalization, compliance to therapy.

### Statistical analysis

Data were analysed using SPSS version 17.0 (SPSS Inc., Chicago, Illinois, USA) for descriptive and frequency results and presented as median and range for quantitative variables and percentiles for categorical variables. Significance was defined as *P* value < 0.05. Comparisons between the two groups used the Fisher’s exact test for categorical variables and the Mann-Whitney U test for quantitative variables. χ2 test was used to determine patients’ acceptance of the products (Actitan F and SOR) we tasted.

## Results

**Fig. 2:**
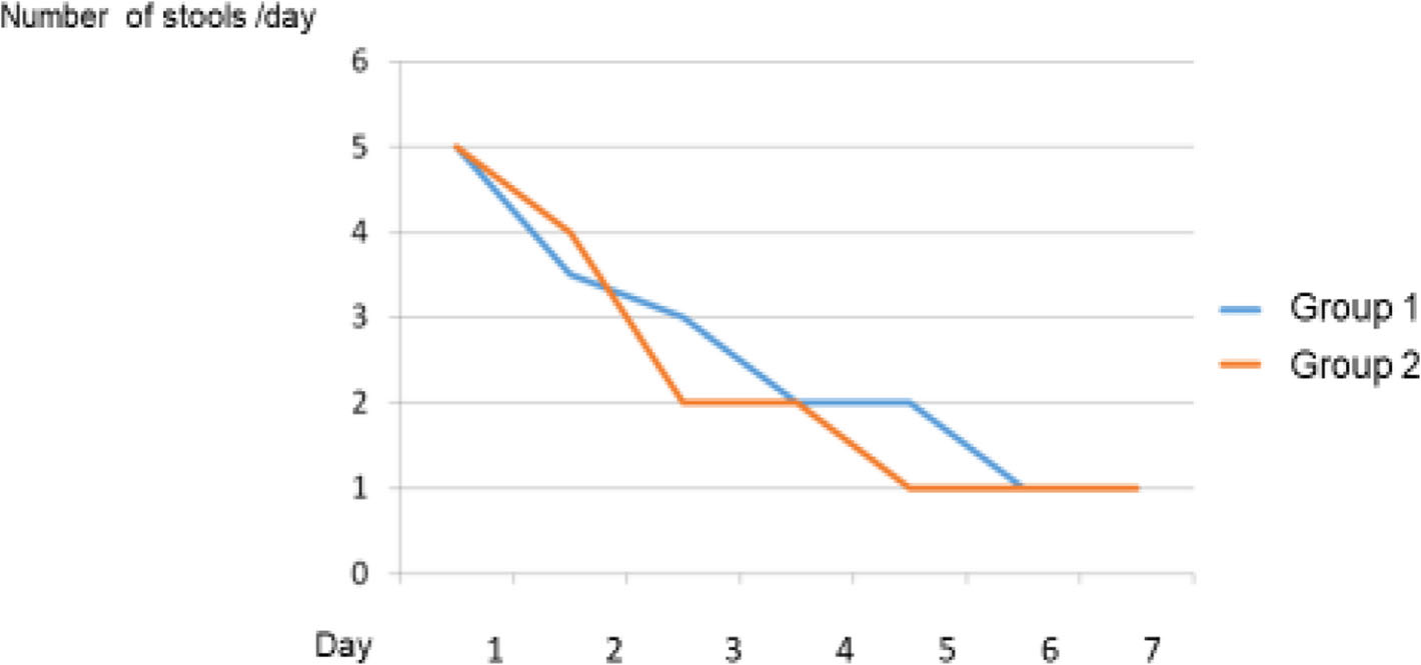
Primary Endpoint: Bowel movements/ day

All patients completed the study. Socio-demographic and clinical characteristics of the study population are showed in Table 1. At baseline, a mean of defecation/daily was 5 in both groups (ranged from 3 to 8 in Group 1 and from 2 to 10 in Group 2) (*p* = 0.63). The median number of stools after 24 h was 3.5 in Group 1 and 4 in Group 2. The difference in the number of stools the first day of treatment between the two groups was not statistically significant (*p* = 0.4); however, the difference between the number of stools at baseline and after 24 h of treatment was significant (p = < 0.0001) in the group treated with Actitan F. After 24 h of treatment, we found that the most of children (23/30, 76%) in Group 1, by their own choice, interrupted the assumption of SOR and continued to take only Actitan F. At the end of treatment, in Group 1 the primary endpoint was reached after 5 days while in Group 2 after 4 days, this difference was not statistically significant (*p* = 0.48) (Fig. 2). We also showed improved stool consistency from baseline to 24 h in the two groups, from liquid stools in 89.7 and 86.7% for Group 1 and Group 2, respectively, to 60 and 73.3% (*p* = 0.34). In particular, regarding stool consistency, we found that at day 6 in Group 1 76.9% of patients reported formed stools compared with 50% in Group 2 (*p* = 0.028). The presence of vomiting at baseline was 73.3% (22 children of 30) for Group 1 and 43.3% (13 children of 30) for the Group 2; at 24 h vomiting was present in 46.6% of the Group1 and 16% of the Group 2 with no statistical significant difference. Bloody diarrhoea was found in only 10 and 6% of patients at baseline for Group 1 and Group 2, respectively; at the end of treatment, no patient presented blood in the stool in both groups. As for weight and fever, we did not find any statistically significant difference between the groups. No one was hospitalized.

**Table 1:** Demographics and Clinical characteristics at baseline

	Overall (*N*=60)	Group 1 (*N*=30)	Group 2 (*N*=30)	*p*-value
Median age (range), years	3.1 (0.3–12.7)	4.1 (0.5–12.1)	2.7 (0.3–12.7)	0.23
Starting date of diarrhea before consultation in days (range)	1 (1–4)	1 (1–4)	1 (1–3)	0.32
Median number of stools/day at baseline (range)	5 (2–10)	5 (3–8)	5 (2–10)	0.63
Consistency of stools at baseline (%)				0.35
Watery	81.4%	86.2%	76.7%	
Soft	18.6%	13.8%	23.3%	
Vomiting at baseline (median and range)	1.5 (0–23)	3 (0–23)	0.5 (0–7)	0.017
Fever (%)	50.0%	50.0%	50.0%	1.0

### Compliance and patient acceptance of treatment

**Fig. 3:**
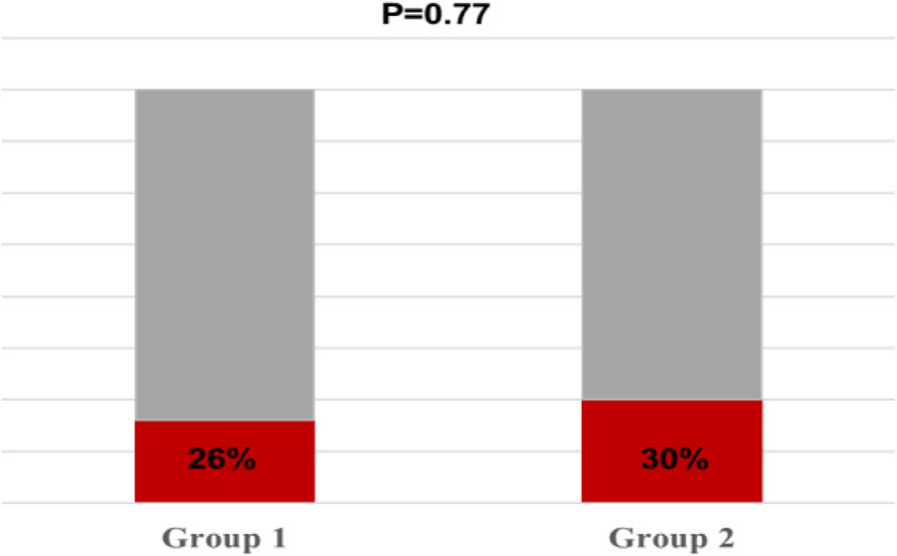
Percentage of patients who complained with flavor of MDSM (Group1) and Oral solution (Group2) (*p* = 0.77)

Concerning compliance with therapy, 8/30 children (26%) in Group 1 and 10/30 (30%) in Group 2 refused to take Actitan F and SOR, respectively (*p* = 0.77) (Fig. 3). Regarding the perception of the treatment efficacy, parents of children in Group 1 evaluated the Actitan F treatment very useful in 50% of cases, while parents of patients in Group 2 evaluated SOR quite effective in 68% of cases, this difference was statistically significant (*p* < 0.0001). Similar trend was present in the perception of improvement after 24 h, where we found a tendency to significance in Group 1 with respect to Group 2 (*p* = 0.07). No adverse events in both groups were reported during the study.

## Discussion

In this pilot study, we investigated the therapeutic efficacy of a medical device made of natural complex substances in the treatment of children with AG. The positive clinical effect exerted by this new product on diarrhoea could be related to the tannins. Their action, indeed, is essentially focused on the intestinal mucosa, favouring its rapid recovery. More specifically, when tannins meet mucous membranes, they aspecifically interact with those membranes by making them more tight and less permeable; this process is called “astringency”. This feature increases protection to the sub-adjacent layers of mucosa from the microorganisms and irritant chemicals [17].

The efficacy and safety of a similar product in treatment of acute diarrhoea in pediatrics have been already demonstrated during the last years. In 2009 Carretero et al. observed a significant decrease in the number of stools and an improvement in stool consistency in patients treated with oral rehydration solution (SOR) and gelatin tannate vs. patients treated only with SOR [18]. Loeb et al. showed that patients receiving a product containing tannate had a normalization of bowel movements, body temperature, weight, and ceased to vomit much faster than those receiving placebo [10].

Plein and colleagues studied the effect of tannate on diarrhoea in patients with Crohn’s disease [19]. The results obtained demonstrated that there was a significant reduction in bowel movement frequency at the end of treatment. Another clinical experience was reported by Ziegenhagen and colleagues, who showed the better efficacy and safety profile of tannin salts versus activated charcoal. Moreover, in the group receiving tannin salts, the frequency of abdominal pain was lower than in the activated charcoal group (50 versus 82%) [20].

Although our study protocol included the administration of Actitan F plus SOR for a week in Group 1 and SOR alone in Group 2, most of the patients in the first Group interrupted the assumption of oral rehydration after the first day of treatment. We can explain this choice considering that after 24 h of combined treatment the reduction in number of stools in Group 1 was significantly different than at baseline (*p* < 0.0001) (5 vs. 3.5 stools/day).

Due to the high perception of symptom improvement reported by parents of children in Group 1 after the first day of treatment, we can speculate that the rapid improvement of clinical condition may have induced parents to stop earlier the oral solution and continue only with Actitan F.

As a matter of fact, our results showing that the mean duration of diarrhea in both groups after 7 days of therapy was not statistically significant, should be interpreted in the light of what we have just described.

Regarding stool consistency, we showed no statistically significant differences between the two groups, however at day 6 of treatment we found that 76.9% of patients in Group 1 reported formed stools compared with 50% in Group 2, this data showed a tendency to significance with a *p* = 0,028, maybe do to the small number of patients enrolled. We can explain this better result on stool consistency comparing to SOR alone, knowing that it has already been demonstrated that tannins and flavonoids exerts their action by restoring the physiological function of the intestine barrier and have astringent properties [17].

Moreover, in our study any adverse event in children were observed in both groups, and this confirms the safety of tannins in the pediatric population. Compliance with administration of Actitan F was good; every patient received 75% of doses according to the patient’s weight. This result confirms a good ease of administration.

In contrast to other studies, we did not perform stool cultures at baseline [21, 22]. Nevertheless, ESPGHAN does not recommend performing stool cultures for acute gastroenteritis in primary healthcare, and we did not aim to evaluate the impact of tannins based on the different aetiologies of diarrhoea.

## Conclusion

Therefore, we can conclude that Actitan F in association with SOR, may offer a safe and cost-effective approach to AG in infants and children. However, further studies and large trials are required. Because this study showed promising results, it would be worthwhile to perform a large randomized control trial to unravel the efficacy of the Actitan F plus SOR in children with AG.

## Abbreviations

AG: Acute gastroenteritis
SOR: standard oral rehydration
